# Nitric Oxide Pathways in Neurovascular Coupling Under Normal and Stress Conditions in the Brain: Strategies to Rescue Aberrant Coupling and Improve Cerebral Blood Flow

**DOI:** 10.3389/fphys.2021.729201

**Published:** 2021-10-22

**Authors:** Cátia F. Lourenço, João Laranjinha

**Affiliations:** ^1^Center for Neuroscience and Cell Biology, University of Coimbra, Coimbra, Portugal; ^2^Faculty of Pharmacy, University of Coimbra, Coimbra, Portugal

**Keywords:** nitric oxide, neurovascular coupling (NVC), cerebral blood flow (CBF), neurodegeneration, oxidative stress, diet, exercise

## Abstract

The brain has impressive energy requirements and paradoxically, very limited energy reserves, implying its huge dependency on continuous blood supply. Aditionally, cerebral blood flow must be dynamically regulated to the areas of increased neuronal activity and thus, of increased metabolic demands. The coupling between neuronal activity and cerebral blood flow (CBF) is supported by a mechanism called neurovascular coupling (NVC). Among the several vasoactive molecules released by glutamatergic activation, nitric oxide (^•^NO) is recognized to be a key player in the process and essential for the development of the neurovascular response. Classically, ^•^NO is produced in neurons upon the activation of the glutamatergic *N*-methyl-D-aspartate (NMDA) receptor by the neuronal isoform of nitric oxide synthase and promotes vasodilation by activating soluble guanylate cyclase in the smooth muscle cells of the adjacent arterioles. This pathway is part of a more complex network in which other molecular and cellular intervenients, as well as other sources of ^•^NO, are involved. The elucidation of these interacting mechanisms is fundamental in understanding how the brain manages its energy requirements and how the failure of this process translates into neuronal dysfunction. Here, we aimed to provide an integrated and updated perspective of the role of ^•^NO in the NVC, incorporating the most recent evidence that reinforces its central role in the process from both viewpoints, as a physiological mediator and a pathological stressor. First, we described the glutamate-NMDA receptor-nNOS axis as a central pathway in NVC, then we reviewed the link between the derailment of the NVC and neuronal dysfunction associated with neurodegeneration (with a focus on Alzheimer’s disease). We further discussed the role of oxidative stress in the NVC dysfunction, specifically by decreasing the ^•^NO bioavailability and diverting its bioactivity toward cytotoxicity. Finally, we highlighted some strategies targeting the rescue or maintenance of ^•^NO bioavailability that could be explored to mitigate the NVC dysfunction associated with neurodegenerative conditions. In line with this, the potential modulatory effects of dietary nitrate and polyphenols on ^•^NO-dependent NVC, in association with physical exercise, may be used as effective non-pharmacological strategies to promote the ^•^NO bioavailability and to manage NVC dysfunction in neuropathological conditions.

## Introduction

**FIGURE 1:**
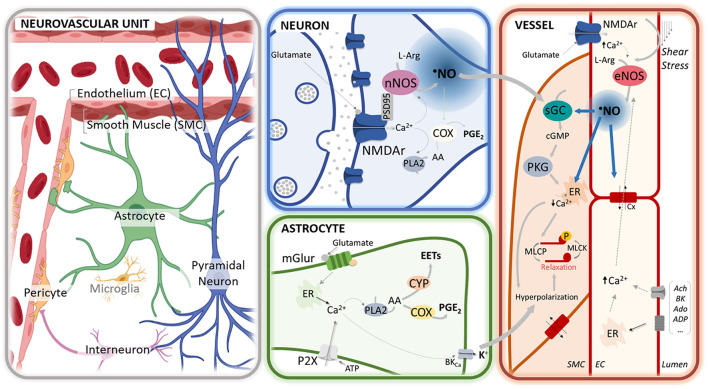
^•^NO-mediated regulation of neurovascular coupling at different cellular compartments of the neurovascular unit. In neurons, glutamate release activates the *N*-methyl-D-aspartate (NMDA) receptors (NMDAr), leading to an influx of calcium cation (Ca^2+^) that activates the neuronal nitric oxide synthase (nNOS), physically anchored to the receptor *via* the scaffold protein PSD95. The influx of Ca^2+^ may further activate phospholipase A_2_ (PLA_2_), leading to the synthesis of prostaglandins (PGE) *via* cyclooxygenase (COX) activation. In astrocytes, the activation of mGluR by glutamate by rising Ca^2+^ promotes the synthesis of PGE *via* COX and epoxyeicosatrienoic acids (EETs) *via* cytochrome P450 epoxygenase (CYP) activation and leads to the release of K + *via* the activation of BK_*Ca*_. At the capillary level, glutamate may additionally activate the NMDAr in the endothelial cells (EC), thereby eliciting the activation of endothelial NOS (eNOS). The endothelial-dependent nitric oxide (^•^NO) production can be further elicited *via* shear stress or the binding of different agonists (e.g., acetylcholine, bradykinin, adenosine, ATP). Additionally, erythrocytes may contribute to ^•^NO release (via nitrosated hemoglobin or hemoglobin-mediated nitrite reduction). At the smooth muscle cells (SMC), paracrine ^•^NO activates the sGC to produce cGMP and activate the cGMP-dependent protein kinase (PKG). The PKG promotes a decrease of Ca^2+^ [e.g., by stimulating its reuptake by sarcoplasmic/endoplasmic reticulum calcium-ATPase (SERCA)] that leads to the dephosphorylation of the myosin light chain through the associated phosphatase (MLCP) and, ultimately to vasorelaxation. Additionally, PKG triggers the efflux of K^+^ by the large-conductance Ca^2+^-sensitive potassium channel (BK_*Ca*_) that leads to cell hyperpolarization. Hyperpolarization is additionally triggered *via* the activation of the inward rectifier potassium channels (Kir) and spread rapidly to adjacent cells *via* gap junctions (Cx). Further, ^•^NO can regulate vasodilation *via* the stimulation of SERCA, modulation of the synthesis of arachidonic acid (AA) derivatives, and regulation of potassium channels and connexins.

The brain is highly dependent on the continuous and dynamically regulated delivery of metabolic substrates to support ongoing neural function. The energy requirements of the brain are expressively high and paradoxically, it has very limited reserves which imply that the blood supply must be finely and timely adjusted to where it is needed the most, which are the areas of increased activity (Attwell and Laughlin, 2001). This process, namely, neurovascular coupling (NVC), is accomplished by a tight network communication between active neurons and vascular cells that involves the cooperation of the other cells from the neurovascular unit (namely, astrocytes, and pericytes) (Attwell et al., 2010; Iadecola, 2017). Despite the extensive investigations and huge advances in the field over the last decades, a clear definition of the mechanisms underlying this process and particularly, the underlying cross-interactions and balance, is still elusive. This is accounted for by the difficulties in measuring the process dynamically *in vivo*, allied with the intrinsic complexity of the process, likely enrolling diverse signaling pathways that reflect the specificities of the neuronal network of different brain regions and the diversity of the neurovascular unit along the cerebrovascular tree (from pial arteries to capillaries). Within such complexity, there is a prevailing common assumption that points to glutamate, the main excitatory neurotransmitter in the brain, as the trigger for NVC in the feed-forward mechanisms elicited by activated neurons. The pathways downstream glutamate may then involve multiple vasoactive molecules released by neurons (*via* activation of ligand-gated cationic channels – iGluRs) and/or astrocytes (via G-coupled receptors activation – mGluRs) (Attwell et al., 2010; Iadecola, 2017; Lourenço et al., 2017a). Among them, nitric oxide (^•^NO) is widely recognized to be an ubiquitous key player in the process and essential for the development of the neurovascular response, as will be discussed in a later section (Figure 1).

A full understanding of the mechanisms underlying NVC is fundamental to know how the brain manages its energy requirements under physiological conditions and how the failure in regulating this process is associated with neurodegeneration. The connection between NVC dysfunction and neurodegeneration is nowadays well-supported by a range of neurological conditions, including Alzheimer’s disease (AD), vascular cognitive impairment and dementia (VCID), traumatic brain injury (TBI), multiple sclerosis (MS), among others (Iadecola, 2004, 2017; Lourenço et al., 2017a; Iadecola and Gottesman, 2019). In line with this, the advancing of our understanding of the mechanisms through which the brain regulates, like no other organ, its blood perfusion may provide relevant cues to forward new therapeutic strategies targeting neurodegeneration and cognitive decline. A solid understanding of NVC is also relevant, considering that the hemodynamic responses to neural activity underlie the blood-oxygen-level-dependent (BOLD) signal used in functional MRI (fMRI) (Attwell and Iadecola, 2002).

In the next sections, the status of the current knowledge on the involvement of ^•^NO in regulating the NVC will be discussed. Furthermore, we will explore how the decrease in ^•^NO bioavailability may support the link between NVC impairment and neuronal dysfunction in some neurodegenerative conditions. Finally, we will discuss some strategies that can be used to counteract NVC dysfunction, and thus, to improve cognitive function.

## Overview on Nitric Oxide Synthesis and Signaling Transduction

### Nitric Oxide Synthases

The classical pathway for ^•^NO synthesis involves a family of enzymes – nitric oxide synthase (NOS) – that catalyzes the oxidation of L-arginine to L-citrulline and ^•^NO, provided that oxygen (O_2_) and several other cofactors are available [nicotinamide adenine dinucleotide phosphate (NADPH), flavin mononucleotide (FMN), flavin adenine dinucleotide (FAD), heme and tetrahydrobiopterin (BH_4_)]. For this to occur, the enzyme must be in a homodimeric form that results from the assembly of two monomers through the oxygenase domains and allows the electrons released by the NADPH in the reductase domain to be transferred through the FAD and FMN to the heme group of the opposite subunit. At this point, in the presence of the substrate L-arginine and the cofactor BH_4_, the electrons enable the reduction of O_2_ and the formation of ^•^NO and L-citrulline. Under conditions of disrupted dimerization, ensured by different factors (e.g., BH_4_ bioavailability), the enzyme catalyzes the uncoupled oxidation of NADPH with the consequent production of superoxide anion (O_2_^–•^) instead of ^•^NO (Knowles and Moncada, 1994; Stuehr, 1999). There are three major members of the NOS family which may diverge in terms of the cellular/subcellular localization, regulation of their enzymatic activity, and physiological function: type I *neuronal NOS* (nNOS), type II *inducible NOS* (iNOS), and type III *endothelial NOS* (eNOS) (Stuehr, 1999). The nNOS and eNOS are constitutively expressed enzymes that rely on Ca^2+^-calmodulin binding for activation. The nNOS and eNOS activity is further regulated both at the transcriptional and post-translational levels and *via* protein-protein interactions (Forstermann and Sessa, 2012). While not exclusively, the nNOS is mainly expressed in neurons where it is intimately associated with glutamatergic neurotransmission. The dominant splice variant of this isoform (nNOSα) possesses an N-terminal PDZ motif that allows the enzyme to bind other PDZ-containing proteins, such as the synaptic density scaffold protein PSD-95. This allows the enzyme to anchor itself to the synaptic membrane by forming a supramolecular complex with the *N*-methyl-D-aspartate receptors (NMDAr), whose activation upon glutamate binding results in Ca^2+^ influx, and ultimately, ^•^NO production. The eNOS isoform is mainly expressed at the endothelium and is critically involved in vascular homeostasis. In the endothelial cells, the eNOS is predominantly localized within the caveolae, forming a complex with caveolin-1 that inhibits its activity. The stretching of the vascular wall, induced by shear stress, results in the dissociation of this complex and allows the enzyme to be activated, either by Ca^2+^-calmodulin binding and/or by PI3K/Akt-mediated phosphorylation of specific serine residues (e.g., 1,177) (Forstermann and Sessa, 2012). Unlike the other two isoforms, iNOS does not rely on Ca^2+^ increases for activation but on the *de novo* synthesis, which occurs predominantly in glial cells following an immunological or inflammatory stimulation. Because iNOS has much lower Ca^2+^ requirements (calmodulin binds with very high affinity to the enzyme even at basal Ca^2+^ levels), it produces ^•^NO for as long as the enzyme remains from being degraded (Knott and Bossy-Wetzel, 2009).

### Nitrate-Nitrite-Nitric Oxide Pathway

In recent years, studies have supported ^•^NO production independent of NOS activity, through the stepwise reduction of nitrate (NO_3_^–^) and nitrite (NO_2_^–^) *via* the so-called nitrate-nitrite-nitric oxide pathway. Viewed as stable end products of ^•^NO metabolism, both NO_3_^–^ and NO_2_^–^ are now recognized to be able to be recycled back into ^•^NO, thereby acting as important ^•^NO reservoirs *in vivo*. NO_3_^–^ and NO_2_^–^ can be consumed in the regular vegetable components of a diet, fueling the nitrate-nitrite-nitric oxide pathway (Rocha et al., 2011; Lundberg et al., 2018). NO_3_^–^ can be reduced to NO_2_^–^ by the commensal bacteria in the gastrointestinal tract and/or by the mammalian enzymes that can acquire a nitrate reductase activity under acidic and hypoxic environments. In turn, the reduction of NO_2_^–^ to ^•^NO can be achieved non-enzymatically *via* a redox interaction with one-electron reductants (e.g., ascorbate and polyphenols) or can be catalyzed by different enzymes (e.g., hemoglobin, xanthine oxidoreductase, and cytochrome P450 reductase). All these reactions are favored by low O_2_ and decreased pH, thereby ensuring the generation of ^•^NO under conditions of limited synthesis by the canonical NOS-mediated pathways which require O_2_ as a substrate (Lundberg et al., 2008). It is also worth mentioning that S-nitrosothiols might serve as downstream ^•^NO-carrying signaling molecules regulating protein expression/function (Chen et al., 2008).

### Nitric Oxide Signal Transduction Pathways

The transduction of ^•^NO signaling may involve several reactions that reflect, among other factors, the high diffusion of ^•^NO, the relative spatial and temporal abundance of the targets, and the relative rate constants with the potential targets. Most of the physiological actions of ^•^NO are promoted by the chemical modification of relevant proteins either *via* nitrosylation or nitrosation [reviewed in Picon-Pages et al. (2019)]. Nitrosylation refers to the reversible binding of ^•^NO to inorganic protein moieties (e.g., iron in heme groups), while nitrosation involves the modification of organic moieties (e.g., thiol groups in cysteine residues), not directly, but intermediated by the species produced upon ^•^NO autoxidation, namely N_2_O_3_. In addition, ^•^NO can react with superoxide anion (O_2_^–•^), yielding peroxynitrite (ONOO^–^), a potent oxidant and nitrating species that conveys the main deleterious actions associated with the ^•^NO signaling (e.g., oxidation and/or nitration of proteins, lipids and nucleic acids) (Radi, 2018).

The best characterized molecular target for the physiological action of ^•^NO is the soluble guanylate cyclase (sGC), a hemeprotein that is frequently and controversially tagged as the classical “^•^NO receptor.” The activation of the sGC by ^•^NO involves the nitrosylation of heme moiety of the enzyme that induces a conformational change, enabling it to catalyze the conversion of guanosine triphosphate (GTP) to the second messenger cyclic guanosine monophosphate (cGMP) (Martin et al., 2005). Nitric oxide may additionally regulate the catalytic activity of sGC by promoting its inhibition *via* nitrosation of critical cysteine residues (Beuve, 2017).

## Nitric Oxide as a Master Player in the Neurovascular Coupling

After being recognized as the endothelial-derived relaxing factor (EDRF) in the late 80s, it did not take long for ^•^NO to be implicated in NVC (Iadecola, 1993). This is not unexpected if we consider that ^•^NO is well suited for such function: it is produced upon glutamate stimulation in the brain, is highly diffusible, and is a potent vasodilator involved in the regulation of the vascular tone.

### Neuronal-Derived ^•^NO Linked to Glutamatergic Neurotransmission

The conventional pathway for ^•^NO- mediated NVC involves the activation of the glutamate-NMDAr-nNOS pathway in neurons. The binding of glutamate to the NMDAr stimulates the influx of [Ca^2+^] through the channel that, upon binding calmodulin, promotes the activation of nNOS and the synthesis of ^•^NO. Being hydrophobic and highly diffusible, the ^•^NO produced in neurons can diffuse intercellularly and reach the smooth muscle cells (SMC) of adjacent arterioles, there inducing the activation of sGC and promoting the formation of cGMP. The subsequent activation of the cGMP-dependent protein kinase (PKG) leads to a decrease [Ca^2+^] that results in the dephosphorylation of the myosin light chain and consequent SMC relaxation [reviewed by Iadecola (1993) and Lourenço et al. (2017a)]. Additionally, ^•^NO may promote vasodilation *via* the stimulation of the sarco/endoplasmic reticulum calcium ATPase (SERCA), *via* activation of the Ca^2+^-dependent K^+^ channels, or *via* modulation of the synthesis of other vasoactive molecules [reviewed by Lourenço et al. (2017a)]. Specifically, the ability of ^•^NO to regulate the activity of critical heme-containing enzymes involved in the metabolism of arachidonic acid to vasoactive compounds suggests the complementary role of ^•^NO as a modulator of NVC *via* the modulation of the signaling pathways linked to mGLuR activation at the astrocytes. ^•^NO has been demonstrated to play a permissive role in PGE_2_-dependent vasodilation by regulating cyclooxygenase activity (Fujimoto et al., 2004) and eliciting ATP release from astrocytes (Bal-Price et al., 2002).

The notion of ^•^NO as a key intermediate in NVC was initially grounded by a large set of studies describing the blunting of NVC responses by the pharmacological NOS inhibition under different experimental paradigms [reviewed (Lourenço et al., 2017a)]. A recent meta-analysis, covering studies on the modulation of different signaling pathways in NVC, found that a specific nNOS inhibition produced a larger blocking effect than any other individual target (e.g., prostanoids, purines, and K^+^). In particular, the nNOS inhibition promoted an average reduction of 2/3 in the NVC response (Hosford and Gourine, 2019). It is recognized that the dominance of the glutamate-NMDAr-NOS pathway in NVC likely reflects the specificities of the neuronal networks, particularly concerning the heterogenic pattern of nNOS expression/activity in the brain. Although nNOS is ubiquitously expressed in different brain areas, the pattern of nNOS immunoreactivity in the rodent telencephalon has been pointed to a predominant expression in the cerebellum, olfactory bulb, and hippocampus and scarcely in the cerebral cortex (Bredt et al., 1990; Lourenço et al., 2014a). Coherently, there is a prevalent consensus for the role of ^•^NO as the direct mediator of the neuron-to-vessels signaling in the hippocampus and cerebellum. In the hippocampus of anesthetized rats, it was demonstrated that the ^•^NO production and hemodynamic changes evoked by the glutamatergic activation in dentate gyrus (DG) are temporally correlated and both dependent on the glutamate-NMDAr-nNOS pathway (Lourenço et al., 2014b). The blockage of either the NMDAr or nNOS also showed to blunt the ^•^NO production and vessels dilation to mossy fiber stimulation in the cerebellar slices (Mapelli et al., 2017).

In the cerebral cortex, ^•^NO has been suggested to act as a modulator rather than a direct mediator of the NVC responses, but this view has been challenged in recent years. Emergent evidence from *ex vivo* approaches indicates that the regulation of vasodilation may diverge along the cerebrovascular tree: at the capillary level, vasodilation seems to be mainly controlled by pericytes *via* an ATP-dependent astrocytic pathway while at the arteriolar level it involves neuronal ^•^NO-NMDAr signaling (Mishra et al., 2016).

### Neuronal-Derived ^•^NO Linked to GABAergic Interneurons

Recent data support that the optogenetic stimulation of nNOS positive interneurons can promote central blood flow (CBF) changes in the somatosensory cortex comparable to those evoked by whiskers stimulation on awake and behaving rodents (Krawchuk et al., 2020; Lee et al., 2020). The implication of the GABAergic interneurons in NVC has been previously demonstrated, both in the cerebellum and somatosensory cortex (Cauli et al., 2004; Rancillac et al., 2006). Also, in the hippocampus, parvalbumin GABAergic interneurons are suggested to drive, *via* ^•^NO signaling, the NVC response to hippocampus-engaged exploration activities with impact in the neurogenesis in the *dentate gyrus* (Shen et al., 2019). The involvement of GABAergic interneurons in neurovascular regulation is not unexpected as some of them have extended projections in close contact with arterial vessels and secrete diverse molecules with vasoactive properties which are able to modulate the vascular tone (e.g., ^•^NO, vasopressin, and NPY) (Hamel, 2006). A novel and striking hypothesis suggest that nNOS-expressing neurons can control vasodilation independent of neural activities. The optogenetic activation of NOS-positive interneurons regulates CBF without detectable changes in the activity of other neurons (Echagarruga et al., 2020; Lee et al., 2020). The activation of GABAergic interneurons has further been shown to promote vasodilation while decreasing neuronal activity; this occurring independently of ionotropic glutamatergic or GABAergic synaptic transmission (Scott and Murphy, 2012; Anenberg et al., 2015). The hypothesis stating that evoked CBF is dynamically regulated by different subsets of neurons, some independently of neuronal activity, calls into question the linearity of the correlation between the net ongoing neuronal activity and CBF changes and raises concerns regarding the interpretation of functional MRI (fMRI) data.

### Endothelial-Derived ^•^NO Linked to Glutamatergic Neurotransmission

As for the systemic vascular network, endothelial-derived ^•^NO has also been implicated in the regulation of CBF. Endothelial cells are able to respond to diverse chemical and physical stimuli by producing, *via* Ca^2+^-dependent signaling pathways, a myriad of vasoactive compounds (e.g., ^•^NO), thereby modulating the vascular tone. Additionally, Ca^2+^ may directly induce the hyperpolarization of the endothelial membrane and adjacent SMC through the activation of Ca^2+^-dependent K^+^ channels (Chen et al., 2014; Guerra et al., 2018). Despite this, the critical requirement of endothelium for the development of a full neurovascular response to neuronal activity only recently started to be valued. Specifically, endothelial-mediated signaling stands to be essential for the retrograde propagation of NVC-associated vasodilation. The discrete ablation of the endothelium was demonstrated to halt the retrograde dilation of pial arteries in response to hindpaw stimulation (Chen et al., 2014). Additionally, in the somatosensory cortex, NVC was shown to be regulated *via* eNOS upon the activation of the purinergic receptors at the endothelium in a mechanism involving a glio-endothelial coupling (Toth et al., 2015). Recent data further pointed to the ability of endothelial cells to directly sense neuronal activity *via* the NMDAr expressed in the basolateral endothelial membranes, thereby eliciting vasodilation *via* eNOS activation (Stobart et al., 2013; Hogan-Cann et al., 2019; Lu et al., 2019). While the precise mechanisms by which the eNOS-derived ^•^NO shape NVC response is still to be defined, eNOS activation is suggested to contribute to the local but not to the conducted vasodilation, the latter being associated with K^+^-mediated hyperpolarization (Lu et al., 2019). Yet, it is proposed that ^•^NO-dependent vasodilation may be also involved in a slower and shorter-range retrograde propagation cooperating with the faster and long-range propagation mediated by endothelial hyperpolarization (Chen et al., 2014; Tran et al., 2018). Of note, ^•^NO can modulate the activity of connexins at the gap junctions to favor the propagation of the hyperpolarizing current upstream to the feeding vessels (Kovacs-Oller et al., 2020). Additionally, vascular-derived ^•^NO has been pointed to facilitate Ca^2+^ astrocytic signal and was forwarded as an explanation for the late endfoot Ca^2+^ signaling (Tran et al., 2018).

### ^•^NO in the Neurovascular Coupling in Humans

Despite the extensive accumulated evidence for the involvement of ^•^NO in the NVC in animal models, these studies have only been applied to humans recently. By addressing the hemodynamic response to visual stimulation, Hoiland and coworkers provided the first demonstration for the involvement of ^•^NO in the NVC in humans *via* modulation by a systemic intravenous infusion of the non-selective competitive NOS inhibitor L-NMMA (Hoiland et al., 2020). The authors proposed a two-step signaling mechanism for the NVC in humans translated in a biphasic response with the first component being attributed to the NOS activation elicited by glutamatergic activation. They hypothesized that ^•^NO may be further involved in the second component of the hemodynamic response *via* erythrocyte-mediated signaling (either by releasing ^•^NO from nitrosated hemoglobin or by mediating NO_2_^–^ reduction) (Hoiland et al., 2020).

## Neurovascular Dysfunction in Neurodegeneration – Focus on Alzheimer’s Disease

The tight coupling between neuronal activity and CBF is crucial in supporting the functional integrity of the brain, by both providing the essential metabolic substrates for ongoing neuronal activities and by contributing to the clearance of the metabolic waste byproducts. Disturbances of the mechanisms that regulate CBF, both under resting and activated conditions, can therefore critically impair neural function. Coherently, a robust amount of data support neurovascular dysfunction implicated in the mechanisms of neurodegeneration and cognitive decline associated with several conditions, including aberrant brain aging, AD, VCID, and TBI, among others [reviewed by Zlokovic (2011), Lourenço et al. (2017a), Sweeney et al. (2018), and Moretti and Caruso (2020)]. A large amount of clinical studies has been focused on AD, for which the regional CBF changes were described to follow a stepwise pattern along the clinical stages of the disease in connection with a cognitive decline (Wierenga et al., 2012; Leeuwis et al., 2017; Mokhber et al., 2021). Alongside, both patients with mild cognitive impairment and AD displayed decreased hemodynamic responses to neuronal activation (memory encoding tasks) (Small et al., 1999; Xu et al., 2007). Interestingly, a retrospective neuroimaging analysis of healthy subjects and patients with mild cognitive impairment and AD suggested that vascular abnormalities are early events, preceding the changes in Aβ deposition, functional impairment, and cerebral atrophy (Iturria-Medina et al., 2016). These and other clinical data are strongly supported by an extensive portfolio of studies in animal models of AD that recapitulate the NVC dysfunction observed in patients [(Mueggler et al., 2003; Shin et al., 2007; Rancillac et al., 2012; Lourenço et al., 2017b; Tarantini et al., 2017), reviewed by Nicolakakis and Hamel (2011)]. The latter has also proved to be valuable in providing insights on the mechanisms underpinning NVC dysfunction and their correlation with AD classical pathological hallmarks, namely, Aβ accumulation, tau hyperphosphorylation, and neuronal loss. For instance, both *in vitro* and *in vivo* studies demonstrated that Aβ can reduce the CBF changes in response to vasodilators and neuronal activation (Price et al., 1997; Thomas et al., 1997; Niwa et al., 2000). In turn, hypoperfusion has been demonstrated to foster both the Aβ production and accumulation (Koike et al., 2010; Park et al., 2019; Shang et al., 2019). Simplistically, this points to a vicious cycle that may sustain the progression of the disease. In this cycle, CBF alterations stand out as important prompters. For instance, in the 3xTgAD mice model of AD, the impairment of the NVC in the hippocampus was demonstrated to precede an obvious cognitive dysfunction or altered neuronal-derived ^•^NO signaling, suggestive of an altered cerebrovascular dysfunction (Lourenço et al., 2017b). Also, the suppression of NVC to whiskers stimulation reported in the tau-expressing mice was described to precede tau pathology and cognitive impairment. In this case, the NVC dysfunction was attributed to the specific uncoupling of the nNOS from the NMDAr and the consequent disruption of ^•^NO production in response to neuronal activation (Park et al., 2020). Overall, these studies point to dysfunctional NVC as a trigger event of the toxic cascade leading to neurodegeneration and dementia.

### Oxidative Stress (Distress) – When Superoxide Radical Came Into Play

The mechanisms underpinning the NVC dysfunction in AD and other pathologies are expectedly complex and likely enroll several intervenients through a myriad of pathways, that may reflect both the specificities of neuronal networks (as the NVC itself) and that of the neurodegenerative pathways. Yet, oxidative stress (nowadays conceptually denoted by Sies and Jones as oxidative distress) is recognized as an important and ubiquitous contributor to the dysfunctional cascades that culminate in the NVC deregulation in several neurodegenerative conditions (Hamel et al., 2008; Carvalho and Moreira, 2018). Oxidative distress is generated when the production of oxidants [traditionally referred to as reactive oxygen species (ROS)], outpace the control of the cellular antioxidant enzymes or molecules [e.g., superoxide dismutase (SOD), peroxidases, and catalase] reaching toxic steady-state concentrations (Sies and Jones, 2020). While ROS are assumed to be critical signaling molecules for maintaining brain homeostasis, an unbalanced redox environment toward oxidation is recognized to play a pivotal role in the development of cerebrovascular dysfunction in different pathologies. In the context of AD, Aβ has been demonstrated to induce excessive ROS production in the brain, this occurring earlier in the vasculature than in parenchyma (Park et al., 2004).

At the cerebral vasculature, ROS can be produced by different sources, including NADPH oxidase (NOX), mitochondria respiratory chain, uncoupled eNOS, and cyclooxygenase (COXs), among others. In this list, the NOX family has been reported to produce more ROS [essentially O_2_^–•^ but also hydrogen peroxide (H_2_O_2_)] than any other enzyme. Interestingly, the NOX activity in the cerebral vasculature is much higher than in the peripheral arteries (Miller et al., 2006) and is further increased by aging, AD, and VCID (Choi and Lee, 2017; Ma et al., 2017). Also, both the NOX enzyme activity level and protein levels of the different subunits (p67phox, p47phox, and p40phox) were reported to be elevated in the brains of patients with AD (Ansari and Scheff, 2011) and AD transgenic mice in correlation with a cognitive decline (Park et al., 2008; Bruce-Keller et al., 2011; Han et al., 2015; Lin et al., 2016). As mentioned earlier, NOS enzymes may produce O_2_^–•^ themselves in their uncoupled state, critically contributing to the decreased BH_4_ bioavailability. Of note, the BH_4_ metabolism is described to be deregulated in AD (Foxton et al., 2007).

**FIGURE 2:**
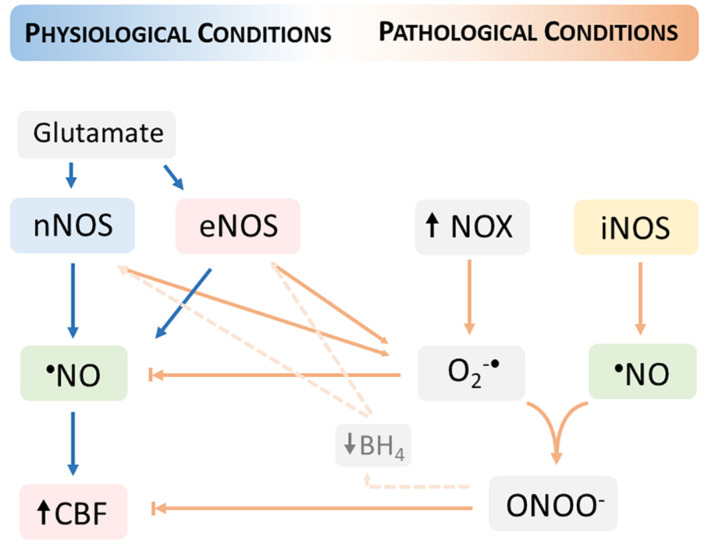
Neurovascular coupling dysfunction in pathological conditions fostered by oxidative distress. The increase in the steady-state concentration of oxygen (O_2_^–•^) produced by the activation of nicotinamide adenine dinucleotide phosphate (NADPH) oxidases (NOX) promotes a decrease in the ^•^NO bioavailability and thus limiting the ^•^NO- mediated cerebral blood flow (CBF) increase. The reaction of ^•^NO with O_2_^–•^ produces peroxynitrite (ONOO)^–^ which additionally contributes to the dysfunction of neurovascular coupling, including by reducing the levels of tetrahydrobiopterin (BH4) and thus promoting the uncoupling of nitric oxide synthases (nNOS and eNOS). The uncoupled NOS produces O_2_^–•^ contributing to increase its steady-state concentration. Under oxidative distress conditions, the inducible NOS (iNOS) supports a sustained ^•^NO production that further reacts with O_2_^–•^.

The reaction of O_2_^–•^ with ^•^NO proceeds at diffusion-controlled rates and is favored by an increased steady-state concentration of O_2_^–•^, providing that ^•^NO diffuses to the sites of O_2_^–•^ formation. This radical-radical interaction has two important consequences for cerebrovascular dysfunction: (1) reduced ^•^NO bioavailability and ensued diminished ^•^NO- mediated vasodilation, and (2) increased peroxynitrite formation and fostering of oxidative distress (Figure 2).

### Decreased ^•^NO Bioavailability and Impaired Neurovascular Coupling: A Central Role for Superoxide Radical

The decrease of ^•^NO bioavailability is translated into inefficient signaling to support the hemodynamic response to neuronal activation and might occur either due to increased scavenging (e.g., by O_2_^–•^) or limited ^•^NO synthesis (e.g., in hypoxia). Thus, the fast reaction of ^•^NO with O_2_^–•^ might be a persuasive reaction at the origins of the ^•^NO decreased bioavailability and NVC dysfunction.

The impact of O_2_^–•^-mediated ^•^NO scavenging in the efficiency of NVC has been addressed in different animal models. In healthy young rats, we were able to mimic the impairment of ^•^NO-mediated NVC observed in the hippocampus of 3xTg-AD mice and aged Fisher 344 rats by using 2,3-dimethoxy-1,4-naphthoquinone (DMNQ), a redox-cycle quinone that triggers the intracellular O_2_^–•^ generation (Lourenço et al., 2017b, 2018). Park and coworkers demonstrated that the attenuation of the CBF changes to whisker stimulation, as observed in aged and AD mice, could be abrogated by both a SOD mimetic (MnTBAP) and a NOX inhibitor (peptide gp91ds-tat) (Park et al., 2007, 2008). In the Tg2576 mice, it was further shown that the genetic ablation of the NOX2 subunit of NADPH oxidase was able to prevent both the NVC dysfunction and spatial memory decline (Park et al., 2008). More recently, the mitochondria-targeted overexpression of catalase has been shown to hamper the age-related NVC dysfunction by preserving the ^•^NO-mediated component of the hemodynamic response (Csiszar et al., 2019).

The ^•^NO synthesis by the NOS enzymes involves the oxidation of L-arginine to L-citrulline, dependent on O_2_. Under conditions of limited O_2_ concentration (e.g., ischemic conditions) and going lower than the K_*M*_ for NOS, the synthesis of ^•^NO by the canonical pathway became limited, and expectedly, the ^•^NO concentration decreases (Adachi et al., 2000).

### Shifting ^•^NO Bioactivity From Signaling Toward Deleterious Actions

As mentioned earlier, the reaction of ^•^NO with O_2_^–•^, yielding ONOO^–^, conveys the major pathway underlying the deleterious actions of ^•^NO, that eventually culminates into neurodegeneration (Radi, 2018). This pathway is largely fueled by the activity of iNOS, an isoform much less dependent on Ca^2+^ concentration and capable to sustain a continuous ^•^NO production, thereby producing a much larger amount of ^•^NO relative to the constitutive isoforms (Pautz et al., 2010). The ONOO^–^ formed can oxidize and nitrate several biomolecules, including proteins. Specifically, the nitration of the tyrosine residues of proteins, resulting in the formation of 3-nitrotyrosine (3-NT), may irreversibly impact signaling pathways (either by promoting a loss or a gain of function of the target protein) (Radi, 2018).

A large body of evidence supports the enhanced 3-NT immunoreactivity in the brains of AD patients and rodent models, as well as the nitration and oxidation of several relevant proteins [reviewed in Butterfield et al. (2011) and Butterfield and Boyd-Kimball (2019)]. Among them, the mitochondrial isoform of SOD (MnSOD) was reported to occur nitrated in AD (Aoyama et al., 2000), a modification associated with enzyme inactivation (Radi, 2004) and expected increased oxidative distress. Also, tau protein has been demonstrated to be a target for nitration, a modification linked to increased aggregation (Horiguchi et al., 2003). In the 3xTgAD mice with impaired NVC, we detected increased levels of 3-NT and iNOS of the hippocampus (Lourenço et al., 2017b). Peroxynitrite can further impair NVC by altering the mechanisms for vasodilation (e.g., oxidizing BH_4_, inhibiting sGC expression/activity, inactivating prostacyclin) and by promoting structural alterations in the blood vessels [reviewed by Chrissobolis and Faraci (2008) and Lee and Griendling (2008)].

## Improving Nitric Oxide Bioavailability to Hamper Neurovascular Dysfunction

**FIGURE 3:**
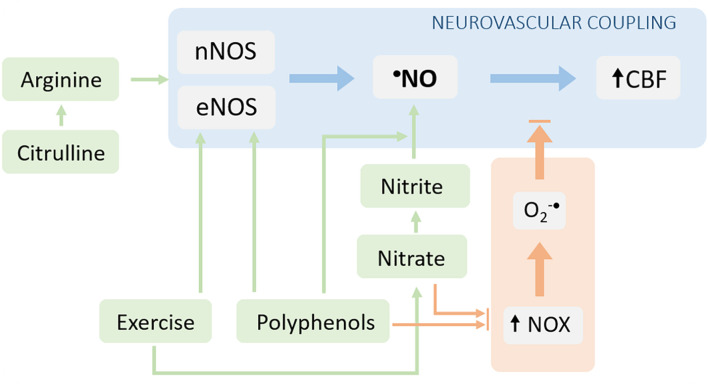
Interventions aiming to target neurovascular coupling dysfunction via reestablishment of ^•^NO bioavailability. The ^•^NO bioavailability can be increased either by stimulating its production (blue arrows) or by decreasing its degradation (orange arrows). The NOS-dependent ^•^NO production can be increased by providing substrates for the enzyme (arginine or its precursor, citrulline) and *via* the post-translational regulation of the enzyme (e.g., signaling pathways stimulated by polyphenols and/or exercise). Additionally, the ^•^NO synthesis can be enhanced *via* the alternative nitrate-nitrite-^•^NO pathway. The limitation of ^•^NO scavenging can be achieved by decreasing the production of superoxide radical (both nitrate/nitrite supplementation and exercise can decrease the activity/expression of NOX enzymes).

From the abovementioned notions, we infer that sustaining ^•^NO bioavailability may have a relevant impact in the NVC, and thus in the neuronal function. Strategies capable to enhance ^•^NO bioavailability may therefore configure attractive approaches to be explored both from a therapeutic standpoint in patients with impaired NVC function (and cognitive decline) or from a prophylactic standpoint in individuals at risk of developing cognitive dysfunctions linked to neurovascular alterations. In line with this, several strategies have been tested, both in animal models and humans, targeting the promotion of ^•^NO synthesis and/or the limitation of its scavenging by the ROS [reviewed by Lundberg et al. (2015)]. Some of the most promising strategies involving non-pharmacological intervention are discussed below (Figure 3).

### Arginine and Citrulline

In the past two decades, both arginine (NOS substrate) and its precursor, citrulline, received a lot of attention for their potential to increase ^•^NO-dependent signaling, being currently accepted that citrulline is more efficient than arginine as a precursor for ^•^NO synthesis (Bahadoran et al., 2021). Citrulline, but not arginine, has been demonstrated to improve the acetylcholine-induced retinal vasodilation in diabetic rats (Kurauchi et al., 2017). It also has been shown to mitigate the cognitive dysfunction linked to transient brain ischemia in mice (Yabuki et al., 2013) and to improve the CBF response associated with spreading depolarization events in rats (Kurauchi et al., 2017). Yet, in patients with mitochondrial encephalomyopathy with lactic acidosis and stroke-like episodes (MELAS) syndrome, oral treatment with L-Arginine was able to restore the NVC response to the visual cortex stimulus (Rodan et al., 2020).

### Nitrite and Nitrate

More recently, much focus has been paid to both NO_2_^–^ and NO_3_^–^ anions as relevant biological precursors of ^•^NO, capable to support ^•^NO-dependent mechanisms, including vasodilation, *via* the NO_3_^–^-NO_2_^–^-^•^NO pathway (Lundberg et al., 2008). In line with this, different pieces of evidence provide support for the benefits of NO_2_^–^ and NO_3_^–^, particularly in the cardiovascular system (Lundberg et al., 2015). Additionally, acute nitrite was demonstrated to enhance basal CBF in rats (Rifkind et al., 2007) and to improve the hemodynamic response to somatosensory activation under conditions of NOS inhibition (Piknova et al., 2011). In essence, NO_2_^–^ is now recognized as a regulator of hypoxic signaling in mammalian physiology through the formation of ^•^NO (Rocha et al., 2011; Lundberg et al., 2018), and human studies support the application of NO_2_^–^ to alleviate cardiovascular dysfunctions and as a supplement for healthy arterial aging (Lundberg et al., 2018). NO_3_^–^, which can be obtained from diets, salt supplementation, or endogenous ^•^NO oxidation, is also proposed to increase both cerebral blood perfusion and cognitive performance in humans, but the overall evidence is still not conclusive (Clifford et al., 2019). This may be justified by the short duration of the interventions and the health status of the subjects. Magnetic resonance imaging studies in an elderly human population exposed to an increased intake of NO_3_^–^-rich foods showed that a 2-day intervention with dietary NO_3_^–^ had no effect on the global CBF but enhanced the CBF in the deep white matter in the frontal lobe, suggested to be involved in executive functioning (Presley et al., 2011). Moreover, in healthy adults, a single dose of beetroot juice (containing 5.5 mmol NO_3_^–^) was demonstrated to modulate their CBF response to task performance in the frontal cortex assessed by near-infrared spectroscopy and to improve their performance to a serial 3 s subtraction task (Wightman et al., 2015). Also, intrathecal levels of NO_3_^–^ in patients with vascular dementia are inversely correlated to the degree of intellectual impairment (Tarkowski et al., 2000). In the cardiovascular and renal systems, dietary NO_3_^–^ was able to attenuate oxidative distress by inhibiting NOX expression/activity, conveying an additional mechanism by which NO_3_^–^ supplementation could improve ^•^NO bioavailability (Carlstrom and Montenegro, 2019).

### Polyphenols

Found predominantly in fruits and vegetables, polyphenols are another set of bioactive compounds recognized to improve ^•^NO bioavailability, thereby providing positive health benefits (Oak et al., 2018). For decades, explored for their antioxidant capacity associated with the free radical scavenging, polyphenols are recognized to directly modulate the redox-sensitive processes *in vivo* (Fraga et al., 2019). Moreover, polyphenols are known to stimulate ^•^NO production in the endothelial cells *via* the phosphorylation of eNOS mediated by both PI3K/Akt and p38 MAPK signaling pathways. Several polyphenols were also described to modulate the redox status of the cellular environment *via* the activation of the nuclear factor (erythroid-derived 2)-like 2 (Nrf2), a transcription factor needed for anti-oxidant response element gene (ARE) expression, leading to the expression of several antioxidant enzymes (e.g., SOD). Interestingly, they have also been demonstrated to downregulate the expression of NOX enzymes (Forte et al., 2016; Fraga et al., 2018). Furthermore, as mentioned above, polyphenols are also capable to promote the reduction of NO_2_^–^ to ^•^NO, particularly under acidic environments (Rocha et al., 2009). Polyphenols accompany NO_2_^–^ and NO_3_^–^ in the vegetables consumed in the human diet and mounting evidence from epidemiological and randomized controlled trials support their beneficial effects in preventing age-associated cognitive decline (Fraga et al., 2019). The ability of some polyphenols to improve NVC has also been demonstrated by some clinical and pre-clinical studies. For instance, resveratrol, a polyphenol found in grape seeds, was able to rescue the hemodynamic responses to neuronal and endothelial activation in aged mice, occurring along with downregulation of the NADPH oxidase and decreased 3-NT levels (Toth et al., 2014). In humans, a similar beneficial effect was demonstrated in type 2 diabetic patients and postmenopausal women. In diabetic patients, a single dose of resveratrol enhanced their performance on a multi-tasking test battery and the blood flow velocity in the middle cerebral artery to this cognitive stimulus (Wong et al., 2016). In postmenopausal women, a long-term treatment with resveratrol significantly increased their cognitive performance and NVC response as compared with their basal condition (Thaung Zaw et al., 2020). Cocoa flavonoids were also demonstrated to improve NVC in humans, increasing the BOLD fMRI response to cognitive tasks in healthy subjects (Decroix et al., 2016) and type 1 diabetic patients (Decroix et al., 2019).

### Exercise

Regular physical exercise is widely recognized to have a substantial neuroprotective impact on brain function, contributing to restore and maintain cognitive performance (Alkadhi, 2018), and amending several cardiovascular risk factors (e.g., diabetes, hypertension) (Kokkinos and Myers, 2010). In particular, exercise is suggested to promote the upregulation of eNOS expression and activity *via* activation of Akt-dependent signaling by temporally increasing shear stress (Chistiakov et al., 2017). Shear stress has also been shown to stimulate the expression of Nrf2 and thus, the upregulation of antioxidant defenses (Warabi et al., 2007). Also, exercise is suggested to enhance NO_2_^–^ plasmatic levels similar to dietary NO_3_^–^ (Lundberg et al., 2015). The evidence for the beneficial effect of exercise in the CBF and brain functioning is overwhelmingly convincing both in humans and experimental animal models (Ogoh and Ainslie, 2009; Smith and Ainslie, 2017; Trigiani and Hamel, 2017). Recently, physical exercise has been demonstrated to alter the white matter pathology and hemodynamic response to whiskers stimulation in a VCID mouse model (Trigiani et al., 2020). Also, in an AD mouse model, exercise training was demonstrated to reduce cerebrovascular dysfunction via enhancing the P2Y2-eNOS signaling (Hong et al., 2020).

## Conclusion

The NVC is a crucial pathway supporting both the structural and functional integrity of the brain. The diffusible vasodilator, ^•^NO, is widely recognized to be a key player in the intricate communication between the neurovascular unit cells required to supply energetic substrates. The canonical pathway for ^•^NO-mediated NVC involves the NMDAr-mediated activation of the nNOS at the neurons and the stimulation of the sGC at the SMC. Additionally, the ^•^NO produced by different sources (interneurons, endothelial cells, erythrocytes) and modulating other signaling pathways likely converge to assure the proper development of the neurovascular response. The failure in this process is linked to neurodegeneration in different pathological conditions and may be fostered by the changes in the redox environment toward oxidation that decreases the ^•^NO bioavailability and subverts its bioactivity. Diet is established among the most relevant adjustable variables of human health in modern societies. Considering that WHO recognizes cognitive impairment and dementia associated with aging as one of the major public health challenges of our time (World Health Organization [WHO], 2012), a more comprehensive understanding of how the different aspects of lifestyle and diet affect neural function and consequent cognitive performance is an imperative need. In line with this, the potential modulatory effects of dietary NO_3_^–^ and polyphenols on ^•^NO-dependent NVC, in association with physical exercise, may be used as effective non-pharmacological strategies to promote the ^•^NO bioavailability and thereby to manage NVC dysfunction in neuropathological conditions.

## Author Contributions

CL and JL discussed the content of the review and approved the final version of the manuscript. CL preformed the literature research, draft the manuscript, and draw the figures. JL edited and revised the manuscript. All authors contributed to the article and approved the submitted version.

## Conflict of Interest

The authors declare that the research was conducted in the absence of any commercial or financial relationships that could be construed as a potential conflict of interest.

## Publisher’s Note

All claims expressed in this article are solely those of the authors and do not necessarily represent those of their affiliated organizations, or those of the publisher, the editors and the reviewers. Any product that may be evaluated in this article, or claim that may be made by its manufacturer, is not guaranteed or endorsed by the publisher.
